# Entrapment of Common Peroneal Nerve by Surgical Suture following Distal Biceps Femoris Tendon Repair

**DOI:** 10.1155/2016/7909805

**Published:** 2016-09-15

**Authors:** Aki Fukuda, Akinobu Nishimura, Shigeto Nakazora, Ko Kato, Akihiro Sudo

**Affiliations:** ^1^Department of Orthopaedic Surgery, Suzuka Kaisei Hospital, 112 Kou, Suzuka, Mie 513-8505, Japan; ^2^Department of Orthopaedic and Sports Medicine, Mie University Graduate School of Medicine, Mie, Japan; ^3^Department of Orthopaedic Surgery, Mie University Graduate School of Medicine, Mie, Japan

## Abstract

We describe entrapment of the common peroneal nerve by a suture after surgical repair of the distal biceps femoris tendon. Complete rupture of the distal biceps femoris tendon of a 16-year-old male athlete was surgically repaired. Postoperative common peroneal nerve palsy was evident, but conservative treatment did not cause any neurological improvement. Reexploration revealed that the common peroneal nerve was entrapped by the surgical suture. Complete removal of the suture and external neurolysis significantly improved the palsy. The common peroneal nerve is prone to damage as a result of its close proximity to the biceps femoris tendon and it should be identified during surgical repair of a ruptured distal biceps femoris tendon.

## 1. Introduction

Hamstring injury is one of the most frequent injuries in a variety of sports activities. Most hamstring injuries are strain, and a complete rupture of the distal part of biceps femoris tendon is extremely rare. Iatrogenic peripheral nerve injury is one of the perioperative complications and is caused by overstretching, prolonged compression, or direct trauma. We describe rare entrapment of the common peroneal nerve by a suture after surgical repair of a ruptured distal biceps femoris tendon.

## 2. Case Report

**Figure 1:**
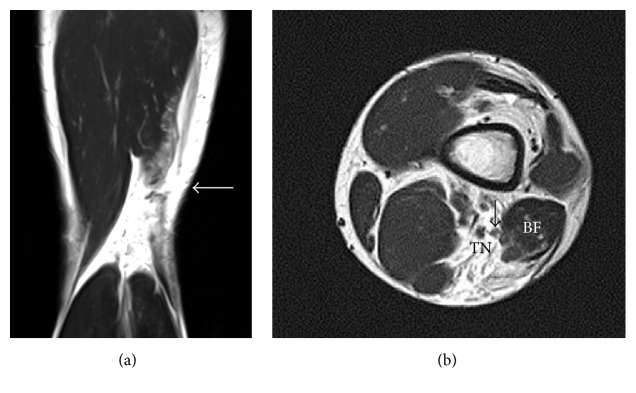
T2-weighted coronal magnetic resonance image of left thigh. Complete rupture of distal biceps femoris tendon at the musculotendinous junction (a). T2-weighted axial magnetic resonance image of left thigh shows close relationship between common peroneal nerve (arrow) and biceps femoris muscle (b). BF, biceps femoris muscle; TN, tibial nerve.

**Figure 2:**
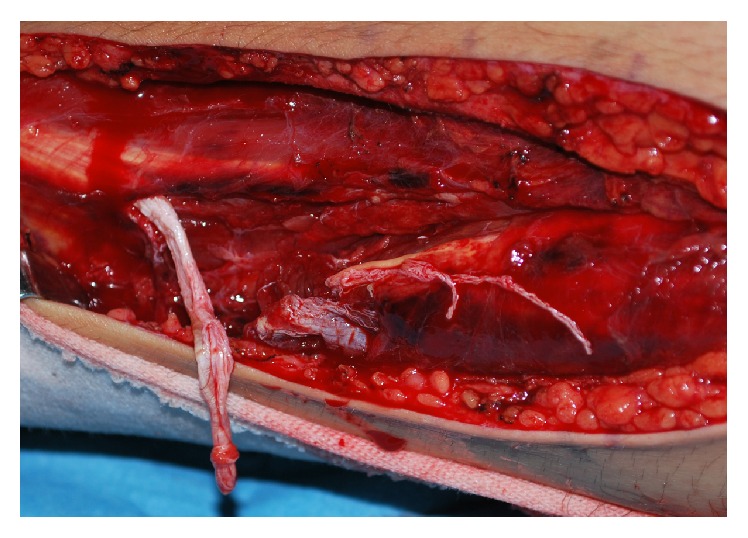
Intraoperative photograph of left leg. Distal biceps femoris tendon is completely ruptured at musculotendinous junction.

A 16-year-old male runner fell forward over an outstretched left leg during a race. He felt a popping sensation with his left hip in deep flexion and the left knee in forced extension and a sharp pain in the posterolateral aspect of the left distal thigh. He could not bear weight on the affected leg, which prevented him from walking and he was immediately transferred to our hospital. He had no history of other trauma, chronic disease, or use of corticosteroids. A clinical examination revealed swelling and tenderness at the posterolateral aspect of the left distal thigh. The ridge formed by the left biceps femoris tendon was distinctly deficient to both visual inspection and palpation. Active extension or flexion of the left knee was prevented by severe pain. Ligamentous instability of the knee was not evident and the left knee appeared normal on plain radiography images. T2-weighted magnetic resonance images of the left thigh revealed a loss of continuity of the distal biceps femoris tendon at the musculotendinous junction surrounded by a hematoma with high signal intensity (Figure 1). Isolated rupture of the distal part of the left biceps femoris tendon was diagnosed and surgical exploration proceeded under lumbar spinal anaesthesia. A lateral incision over the belly of the biceps femoris revealed that the biceps femoris tendon had completely ruptured at the musculotendinous junction (Figure 2). The tear was repaired using a modified Kessler method with strong nonabsorbable sutures.

**Figure 3:**
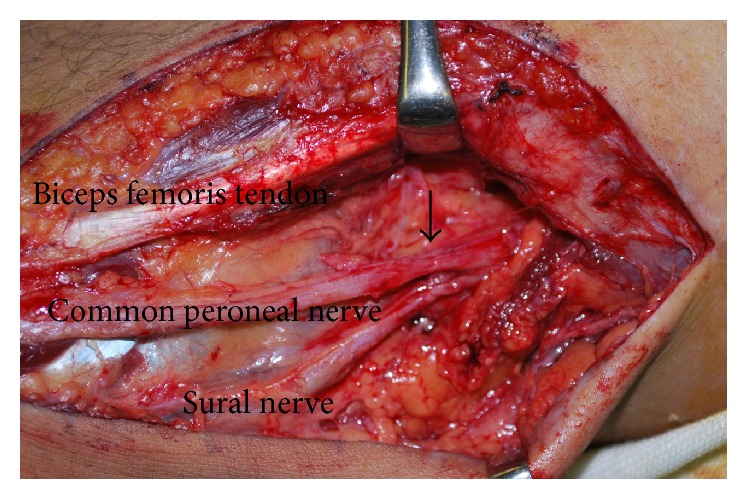
Photograph at reoperation. Hourglass-like constriction of common peroneal and sural nerve (arrow).

The knee was postoperatively immobilized in a plaster cast at 30° of flexion. However, the patient described numbness and sensory loss over the anterior aspect of the left foot and dorsiflexion of the foot was weak on the day after surgery. Common peroneal nerve palsy at the fibular head due to compression by the plaster cast was diagnosed and the cast was removed to decompress the nerve. However, this did not result in any neurological improvement. Surgical assessment was applied 2 months after the common peroneal palsy occurred. Reexploration findings showed complete repair of the ruptured tendon, but the common peroneal and sural nerves were entrapped by the surgical suture, which had also penetrated the common peroneal nerve. Both nerves were obviously constricted after removal of the surgical suture (Figure 3). Both nerves were externally neurolysed and scar tissue was removed. Motor function completely recovered 15 months later, but some residual numbness in the lower left leg persisted.

## 3. Discussion

Complete rupture of the distal biceps femoris tendon is extremely rare and only a few cases have been described in the literature [1–5]. The mechanism of injury in our patient was a combination of deep hip flexion and forced knee extension, which caused a complete rupture of the distal part of the biceps femoris tendon. Most hamstring injuries are strains that can be treated without surgery. However, surgery can clinically improve ruptured distal biceps femoris tendons. Lempaine reported that eight of nine distal biceps femoris tendon injuries at the musculotendinous junction were chronic and surgically treated between 1.5 and 15 (average, 5.4) months after injury because of unsatisfactory outcomes of conservative nonsurgical therapy [6]. However, all of these patients returned to their preinjury level of performance at an average of 3.5 months after surgery. The biceps femoris muscle is a powerful flexor and an important dynamic stabilizer of the knee. Biomechanical studies have shown a 30%–85% decrease in flexion after resection of the biceps femoris tendon [1]. These results suggest that surgery effectively minimizes loss of muscle function and strength after injury to the distal biceps femoris tendon.

**Figure 4:**
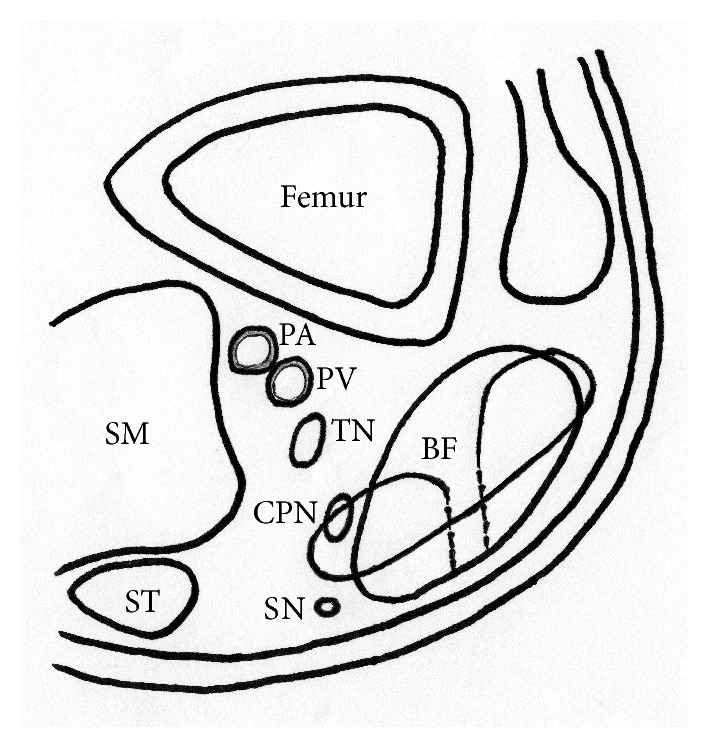
Schema of common peroneal nerve entrapped by surgical suture. BF, biceps femoris muscle; CPN, common peroneal nerve; PA, popliteal artery; PV, popliteal vein; SM, semimembranosus muscle; SN, sural nerve; ST, semitendinosus muscle; TN, tibial nerve.

Perioperative iatrogenic peripheral nerve injuries are recognized complications in clinical practice, as they arise at a rate of 10% [7]. Common causes of such nerve injuries include overstretching or prolonged compression caused by poor positioning during surgery or cast immobilization. Other causes include direct trauma during injection or surgery [8, 9]. The most common injury sites are the brachial plexus and the accessory nerve, the radial and posterior interosseous nerves, the ulnar nerve, and the common peroneal nerve [7]. Among them, the common peroneal nerve is the most common among iatrogenic nerve injuries of the lower extremities. The common peroneal nerve originates from the sciatic nerve, passes posteriorly and laterally to the biceps femoris muscle in the popliteal fossa, crosses the head of the fibula, and descends the lower leg. Therefore, the common peroneal nerve is prone to injury, particularly at the fibular head, due to its superficial location. The common peroneal nerve palsy in our patient was possibly due to compression at the fibular head during cast immobilization and direct trauma during surgery. Compression injury at the fibular head, which is the most common location of postoperative nerve palsy, was initially diagnosed and conservatively treated. Most compression injuries recover over time if diagnosed and appropriately managed, but this did not occur in our patient. Surgical assessment 2 months after the initial operation revealed that the common peroneal and sural nerves were entrapped by the surgical suture (Figure 4). The direct injury of the common peroneal nerve in our patient was due to the anatomical relationship between the biceps femoris tendon and the common peroneal nerve. The common peroneal nerve is bound to the biceps femoris muscle by well-defined fascia [10]. The common peroneal nerve runs very deep and dorsal to the surgical site of the biceps femoris tendon rupture at the musculotendinous portion, although it runs superficially around the fibular head. Therefore, the common peroneal nerve is difficult to visualize and thus vulnerable to iatrogenic injury at the musculotendinous junction of the biceps femoris muscle. The symptoms in our patient improved over time, although slight numbness persisted in the left lower leg. Proper diagnosis and early surgical assessment would have probably minimized the neurological disability in our patient.

## 4. Conclusions

We described rare entrapment of the common peroneal nerve by a surgical suture after repair of a ruptured distal biceps femoris tendon. Complete rupture of the distal part of the biceps femoris tendon is extremely rare and it should be treated surgically to minimize loss of muscle function. The common peroneal nerve is prone to injury due to its close proximity to the biceps femoris tendon. Therefore, awareness of the potential risk for nerve injury is important and the common peroneal nerve should be identified during the surgical repair of a ruptured distal biceps femoris tendon.
